# Veratrum Viride as a Remedy in Convulsive Diseases of Children

**Published:** 1876-06

**Authors:** B. Frank Humphreys

**Affiliations:** Hawkins, Texas


### VERATRUM VIRIDE AS A REMEDY IN CONVULSIVE DISEASES OF CHILDREN.

By B. FRANK HUMPHREYS, M. D. Hawkins, Texas.

The successful physician is not the one that uses the greatest variety of remedies or queer and complicated prescriptions, many of which are nothing more than curious combinations of articles which are chemically and therapeutically incompatible. The shot-gun method of giving a "compound," containing some special remedy for each seeming indication, is, to say the least of it, very irrational, and successful as a general mode of practice, and will, in a great measure, pass away before the brighter dawn that is breaking. If many remedies *must be used* in any given case, it would prove more satisfactory, for the most part, to give them in alternation.

We may not need a great number of new remedies—indeed we should not want them, unless better understood than hundreds that have been recently brought to notice, which, when thoroughly tested, are found to be destitute of the wonderful properties ascribed to them, by a class of "progressionists," forsooth, who would turn the world upside down, if all were to heed their folly. It is well for mankind, however, that these visionary prodigies explode, sooner or later, none being deceived but themselves and a certain class of superficial observers.

While we may not need a long catalogue of new remedies, we do need a better knowledge of those we have. Our *materia medica* should be better understood. Every physician should study diligently, to learn for himself the special therapeutic value of each article of the materia medica, and to know, with certainty, its *leading medical properties.* While we would not ignore the truths brought to light by our fathers in medicine, we should discard their errors, and endeavor to commit none greater.

Instead of multiplying the properties of the various articles of the materia medica, it would be better, perhaps, to restrict their employment, using this or that special remedy only when we know it is the best—the most certain to give the desired result.

*Veratrum viride* is a remedy that needs further study. As its properties are better understood, it is sure to rank as one of the leading and most important of our therapeutic resources. Even now, he who has used it most frequeutly, when indicated, prizes it most highly, without any regret of having employed it too often.

Young children are not unfrequently attacked with convulsions, which may be produced by a variety of causes, differing very much from each other. The remedy or remedies which may "act like a charm" in convulsions produced by one cause, may prove destructive if given for paroxysms arising from a directly opposite cause.

In many portions of this State we have our quota of malarial fevers. Some seasons they are very malignant, especially during the latter part of the summer and beginning of autumn; many of the cases are of a congestive type, while the smaller children are frequently affected with violent convulsions.

During the acme of a febrile exacerbation, the child is seized with a convulsive paroxysm, the usual symptoms being present, while there is rarely any pulse; sometimes, however, there can be felt a weak, tremulous motion, barely perceptible. Evidently the convulsion is the result of an unequal circulation. To remove this difficulty is the first step towards a successful treatment.

It is probable that some would hesitate, at first thought, to give veratrum while the little patient was *pulseless*. Nevertheless, it is the special remedy to restore the circulation; and hence the great *anti-spasmodic*, so to speak, on which we may rely with confidence in such an extremity.

A few cases in practice will better illustrate the condition for which the veratrum will prove most successful.

CASE I.

*October 3rd,* 1875.—Was called at 2 p. m., to Minnie Wells, aged $2\frac{1}{2}$ years. She had been unwell a few days. Found the pulse at 160, vibrations not very distinct, rather weak, intermitting one beat in four; fretful, crying, eyes nearly closed; suffering extreme; light yellow coat on tongue; features distressed; cheeks a little flushed; pale around the mouth; alae of the nose strikingly pinched, etc.

Preseribed Norwood's veratrum, one drop every hour or two, as required, for the fever; upon the decline of which the child was to have antiperiodic doses of quinine, and a few powders of calomel. Seeing the child was threatened with convulsions, I prescribed, in addition to the above, chloral hydrate, should it be needed.

Just as I was going to leave, the child had a spasm. This was about 4 p. m. Immediately the chloral was given, in 3 or 4-grain doses, as often as seemed necessary; but notwithstanding the dose was increased, and frequently repeated, the child had a paroxysm every fifteen minutes. In the meantime it was bathed in warm water, and cold packs applied to the head. Thus matters continued until 6 p. m., when the child was brought under the influence of the chloral, and slept one hour. Upon awaking the convulsions returned with greater violence than before. The chloral was now given with but little effect, the fits recurring every fifteen minutes.

Finally, the chloral was pushed in larger and still larger doses, as it seemed to me then as the main hope.

About 10 p. m. the child was brought fully under the in influence of the chloral—and I never desire to see another child in its condition! For one long, long hour, the parents nursed and wept over their only child, that appeared to be dying, so heavy and stertorous was its breathing, while it seemed every moment as though it would suffocate, on account of the choked-up condition of its lungs, from which a peculiar rattling sound was heard at every inspiration, as though the child was *in articulo*

*mortis*. Seeing these alarming effects follow the use of the chloral hydrate, I resolved to give no more.

The child was frequently threatened with convulsions after this, but they were forestalled with veratrum, which was used until a while after midnight. During the latter part of the night, nothing was given to the child but cold water, with cold packs to the head, renewed every few minutes as long as necessary.

At daylight the little patient appeared to be sinking very rapidly, the parents weeping over it as though it were already dead; and indeed it looked very much like a corpse. Here I put the child upon full doses of quinine, and, in a few hours, it was evidently better. The mercury was continued until it had accomplished its purpose. The quinine was kept up for a few days, and the veratrum, to control the circulation when too high.

By careful nursing, the child made a good recovery.

CASE II.

*October 22nd*, 1875.—Was called, at 3 p. m., to Luna Howard, of this place, aged a little over two years, who, having had a light febrile paroxysm, about noon, was seized with a violent spasm at 3 p. m., which continued, without intermission, until 8 p. m., when the paroxysm ceased.

The warm mustard bath and cold applications to the head were used perseveringly, for a time, without any apparent benefit. There was no pulse; respiration exceedingly hurried; eyes set in their sockets; teeth grating, and firmly clenched most of the time.

Veratrum and chloral were given alternately every half hour, until I again noticed the unpleasant effects of the chloral upon respiration, when it was suspended and the veratrum continued, one drop every hour, as the child was very delicate and of the nervous-sanguine temperament.

As soon as the pulse could be counted, it was found to be 180, small and corded. At 6 p. m. the skin became moist and pliant; pulse less frequant; teeth not so firmly clenched; and by 8 p. m., the pulse was nearly normal—the spasm completely subdued.

The child was now put upon quinine and mercury, which course was continued a few days.

Visiting the little patient the next morning, I found it doing quite well, and it made a speedy recovery without further attention.

CASE III.

Johnnie Morris, of this town, about the age of the last case, had been suffering some days with intermittent fever, for which his father medicated him, but without success.

*November 5th*, 1875.—The child had a light chill in the morning, followed by considerable fever, and went into convulsions about 2 p. m. So terrible did the sight appear to the young mother, to see her only child thus distorted and writhing, that she suddenly rushed into my presence, weeping aloud and wringing her hands in despair, and exclaimed, most imploringly, "*Oh! Doctor! my child is dying*—IS DYING! *What shall I do?*" She ran swiftly home, a short distance, and I followed in haste. The grandmother of the child was bathing him in warm water, after which he was wrapped in blankets.

The symptoms were much the same as in the last case.

Immediately I gave him two drops of veratrum and about three grains of chloral hydrate. After this the remedies were given alternately every two hours until relaxation was complete, when the chloral was suspended and the veratrum continued until the fever subsided.

At 9 p. m., the same day, the child was clear of fever, from which time quinine was given freely through the night, and for a few days, together with the usual mercurial course.

He made a quick and complete recovery.

The success, in each of these cases, may be attributed partly to the collateral remedies, and with good reason. Each did its part in establishing convalescence; nevertheless, I think it will be conceded that the veratrum viride was the main agent in arresting the convulsive type of the disease in each case.

Sometimes I have used belladonna, alternated with aconite, (or the two combined), in small doses, frequently repeated, with most satisfactory results. In others, with precisely the same symptoms, such treatment was useless—so I was forced to abandon it and resort to the veratrum.

Many similar cases might be given, but it is unnecessary, as the above are fair examples.

I have known a number of children affected just like those described, who were lost in a few days—sometimes in a few hours—notwithstanding they were under the care of intelligent physicians; who, however, pursued a very different line of treatment from that given in this paper.

Having met with no failure in such cases, where the foregoing, or like treatment, was adopted in time, I have had but one object in writing this article. Should it assist the faithful physician in restoring one of these little patients to the fond embrace of despairing friends, I shall feel fully repaid, and my highest aim will be secured.—*Nashville Medical Journal.*

